# A molecular fragment cheminformatics roadmap for mesoscopic simulation

**DOI:** 10.1186/s13321-014-0045-3

**Published:** 2014-10-04

**Authors:** Andreas Truszkowski, Mirco Daniel, Hubert Kuhn, Stefan Neumann, Christoph Steinbeck, Achim Zielesny, Matthias Epple

**Affiliations:** 1 Inorganic Chemistry and Center for Nanointegration Duisburg-Essen (CENIDE), University of Duisburg-Essen, Essen, Germany; 2 Institute for Bioinformatics and Cheminformatics, Westphalian University of Applied Sciences, Recklinghausen, Germany; 3 CAM-D Technologies, Essen, Germany; 4 GNWI - Gesellschaft fuer naturwissenschaftliche Informatik mbH, Oer-Erkenschwick, Germany; 5 Chemoinformatics and Metabolism, European Bioinformatics Institute (EBI), Cambridge/Hinxton, UK

**Keywords:** Dissipative particle dynamics, Computer simulation, Molecular fragmentation, fSmiles, Fragment smiles, Molecular fragment cheminformatics, Molecular fragment dynamics, Mesoscopic simulation, Peptide representation, Protein representation

## Abstract

**Background:**

Mesoscopic simulation studies the structure, dynamics and properties of large molecular ensembles with millions of atoms: Its basic interacting units (beads) are no longer the nuclei and electrons of quantum chemical ab-initio calculations or the atom types of molecular mechanics but molecular fragments, molecules or even larger molecular entities. For its simulation setup and output a mesoscopic simulation kernel software uses abstract matrix (array) representations for bead topology and connectivity. Therefore a pure kernel-based mesoscopic simulation task is a tedious, time-consuming and error-prone venture that limits its practical use and application. A consequent cheminformatics approach tackles these problems and provides solutions for a considerably enhanced accessibility. This study aims at outlining a complete cheminformatics roadmap that frames a mesoscopic Molecular Fragment Dynamics (MFD) simulation kernel to allow its efficient use and practical application.

**Results:**

The molecular fragment cheminformatics roadmap consists of four consecutive building blocks: An adequate fragment structure representation (1), defined operations on these fragment structures (2), the description of compartments with defined compositions and structural alignments (3), and the graphical setup and analysis of a whole simulation box (4). The basis of the cheminformatics approach (i.e. building block 1) is a SMILES-like line notation (denoted *f*SMILES) with connected molecular fragments to represent a molecular structure. The *f*SMILES notation and the following concepts and methods for building blocks 2-4 are outlined with examples and practical usage scenarios. It is shown that the requirements of the roadmap may be partly covered by already existing open-source cheminformatics software.

**Conclusions:**

Mesoscopic simulation techniques like MFD may be considerably alleviated and broadened for practical use with a consequent cheminformatics layer that successfully tackles its setup subtleties and conceptual usage hurdles. Molecular Fragment Cheminformatics may be regarded as a crucial accelerator to propagate MFD and similar mesoscopic simulation techniques in the molecular sciences.

**Graphical abstract:**
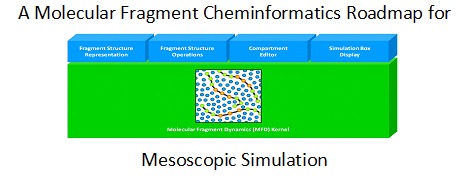
A molecular fragment cheminformatics roadmap for mesoscopic simulation.

**Electronic supplementary material:**

The online version of this article (doi:10.1186/s13321-014-0045-3) contains supplementary material, which is available to authorized users.

## Background

Molecular modelling and simulation aims at (at least) theoretically explaining and (at best) predicting the structures, properties and dynamics of molecules and molecular ensembles. Whereas the fundamental laws of nature are known in principle for nearly a century [1], their practical application required the development of sufficiently fast computing devices in combination with corresponding theoretical approximations - a venture that successfully forged ahead in the last decades as indicated by the 1998 and 2013 Nobel Prizes in chemistry [2],[3]. Due to the exponentially growing computational power - sketched by “Moore’s law” [4] - the frontiers of molecular modelling and simulation could be expanded to successively higher levels of theory as well as to a constantly enlarged size of the chemical entities and ensembles under investigation [5]-[8].

Dissipative particle dynamics (DPD) in particular is a well-established mesoscopic simulation technique to study the structure, dynamics and properties of very large molecular ensembles which may represent millions of atoms. Its basic coarse-grained interacting units (beads) are no longer the nuclei and electrons of quantum chemical ab-initio calculations or the fine-grained atom types of molecular mechanics but appropriate larger molecular shapes which may not necessarily be distinct chemical compounds at all [9]-[11]. The motions of DPD beads follow Newton’s equations of motion where the effective forces are composed of a conservative part due to specific bead-bead pair potentials as well as an additional fluctuating (random) and a dissipative contribution [12]-[15]. The latter two forces act like a thermostat conserving the total momentum and introducing Brownian motion into the system. The DPD technique is designed to obey the Navier-Stokes equations of hydrodynamics and to rigorously sample the canonical ensemble [16]. Molecular fragment dynamics (MFD) is a particular chemical intuitive DPD variant: Its beads are chosen to be specific molecules or molecular fragments where each distinct chemical compound is represented by a specific set of fragments which are connected by harmonic springs in an appropriate manner to describe the intra-compound covalent bonding [17]-[20].

A MFD kernel software for mesoscopic simulation has a simple architecture in principle: It comprises a main loop for a defined number of successive iteration steps to approximately solve the equations of motion. The positions and velocities of all beads are stored in appropriate array structures which represent the corresponding mathematical vectors. All topological information, e.g. the mutual connections between the beads of a specific molecule, is coded with speed- or memory-optimized data structures that represent the corresponding mathematical connection matrices. A simulation input consists of a set of initial bead positions for simulation start, the complete molecular connectivity information and numerous additional parameters that guide the simulation process like the number of simulation steps. The simulation output contains sets of bead positions for different simulation steps as well as corresponding calculated properties. All input or output of a simulation engine is usually provided or generated in form of adequate ASCII files which often comprise hundred thousands of lines. Thus performing a simulation task with a pure kernel software requires a manual setup and interpretation of these ASCII files - a tedious, time-consuming and above all error-prone venture that considerably limits practical usage and application.

A virtue of Cheminformatics is to develop concepts, definitions, data structures, algorithms and toolbox software that allow an efficient and comfortable treatment of chemical entities and ensembles at the man-machine interface [21]-[23]. This study aims at outlining a complete Molecular Fragment Cheminformatics (MFC) roadmap that frames a MFD simulation kernel to tackle the problems sketched above.

## Results and discussion

**Figure 1:**
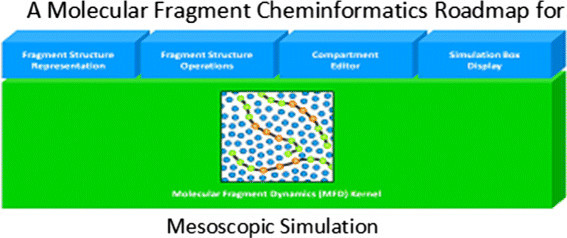
**MFC roadmap.** Building blocks of the MFC layer (blue) above the MFD kernel.

An MFC roadmap consists of four consecutive building blocks above the MFD kernel (see Figure 1): An adequate fragment structure representation (1), a defined set of operations on these fragment structures (2), the description of compartments with defined compositions and structural alignments (3) and the graphical setup and analysis of a whole simulation box (4). These building blocks are outlined in the following.

### Building block 1: Fragment structure representation

**Figure 2:**
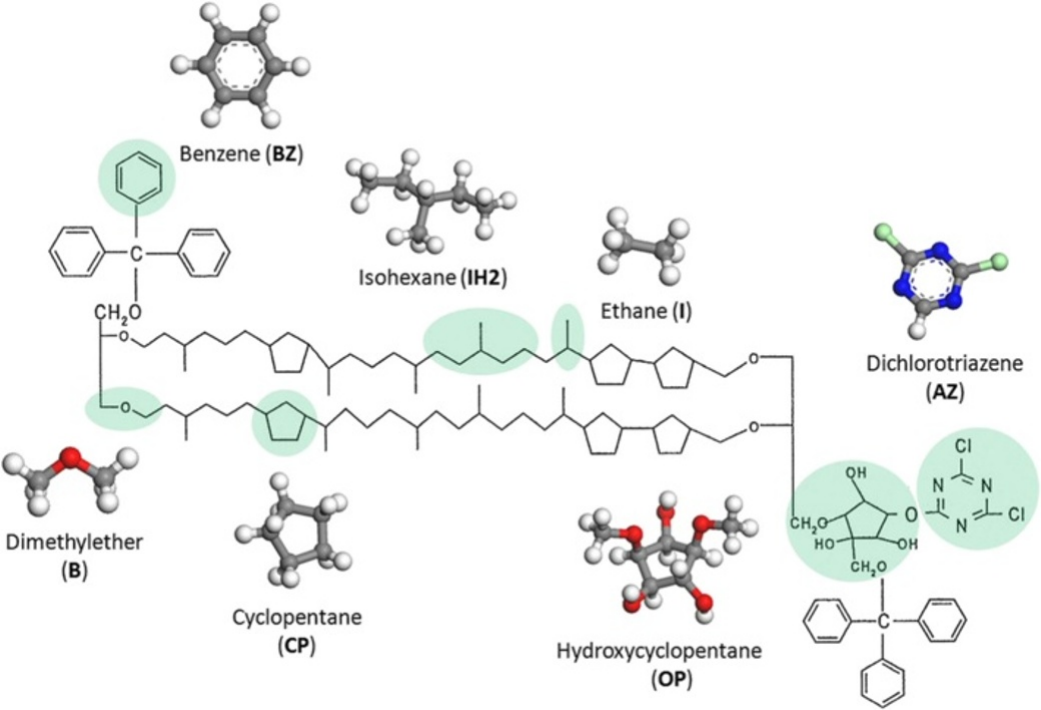
**GDNT lipid fragmentation.** Possible fragments for a GDNT lipid representation.

**Figure 3:**
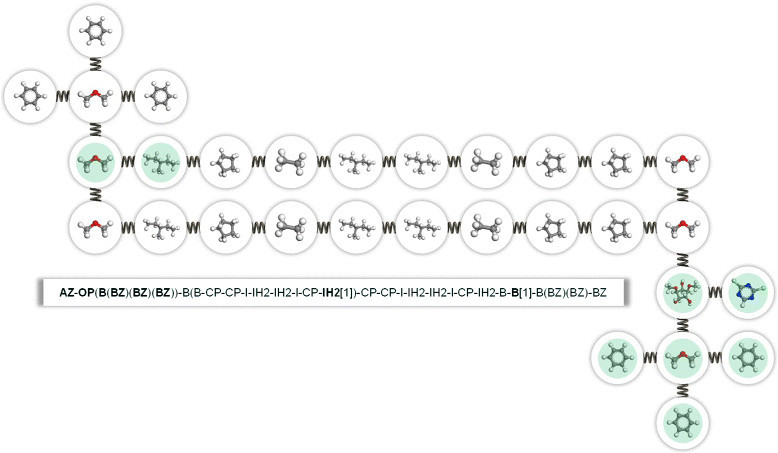
**GDNT lipid fragmentation with** ***f*** **Smiles.** Fragmentation scheme with corresponding *f*SMILES for a GDNT lipid (the fragments highlighted in bold letters correspond to the highlighted fragments in the fragmentation scheme, the fragment connections are illustrated by harmonic springs).

A molecular fragment structure for a chemical compound consists of molecular fragments which are connected by harmonic springs where a single fragment may have multiple connections to other fragments. The fragmentation of a distinct chemical compound is not a unique and objective procedure but only an adequate approximation driven by experience and chemical intuition. As an example, non-ionic polyoxyethylene alkyl ether surfactants with the general formula C_*x*_H_2*x*+1_(OCH_2_CH_2_)_*y*_OH, abbreviated as C_*x*_E_*y*_, may be approximated by *x* linearly connected methane fragments followed by *y* connected dimethyl ether fragments and a terminal methanol fragment [20]. Alternatively, the initial carbon chain could be represented by larger fragments like ethane, propane or butane, e.g. C _10_*E*_4_ may consist of 9 connected methane fragments or of 3 connected propane fragments. Due to the multiple possible connections of a single fragment, a fragment structure may contain branches as well as ring closures: Figure 2 illustrates a possible fragment definition for a glycerol dialkyl nonitol tetraether (GDNT) lipid and Figure 3 shows a complete fragmentation scheme resulting from the chosen fragments. To specifically represent polymers a fragment structure should allow monomer definitions. For orientation/alignment in possible simulation box compartments corresponding tags should be optionally available to be attributed to specific fragments. Last but not least, a fragment structure may contain independent parts, e.g. to describe a protein like human hemoglobin which consists of different chains without any covalent connectivity in between. For a compact molecular fragment structure representation that addresses all the sketched requirements, Appendix A describes a set of nine rules that allow an ASCII string fragment structure line notation. This line notation is chosen to be intuitive and similar to the well-established SMILES representation for atom-based molecular connectivity [24]-[26] and therefore denoted *fragment* SMILES or *f*SMILES. Appendix B shows several examples of *f*SMILES to outline the defined rules. It should be noted that molecular attributes like electric charges or chiral structure are intrinsic properties of the individual fragments and may not be specified in the structure representation, i.e. differently charged states or different enantiomers of a molecular fragment correspond to different fragments where each fragment has a specific charge and a specific stereochemistry. For the C _*x*_*E*_*y*_ surfactants sketched above, a general *f*SMILES would be *(x − 1)Methane-yDME-MeOH* (with Methane: Methane fragment, DME: Dimethyl ether fragment and MeOH: Methanol fragment), e.g. 9Methane-4DME-MeOH9Methane-4DME-MeOH for C _10_*E*_4_. The *f*SMILES with branches and ring closure for a GDNT lipid is given in Figure 3.

#### Fragment structure editors and visualization

**Figure 4:**
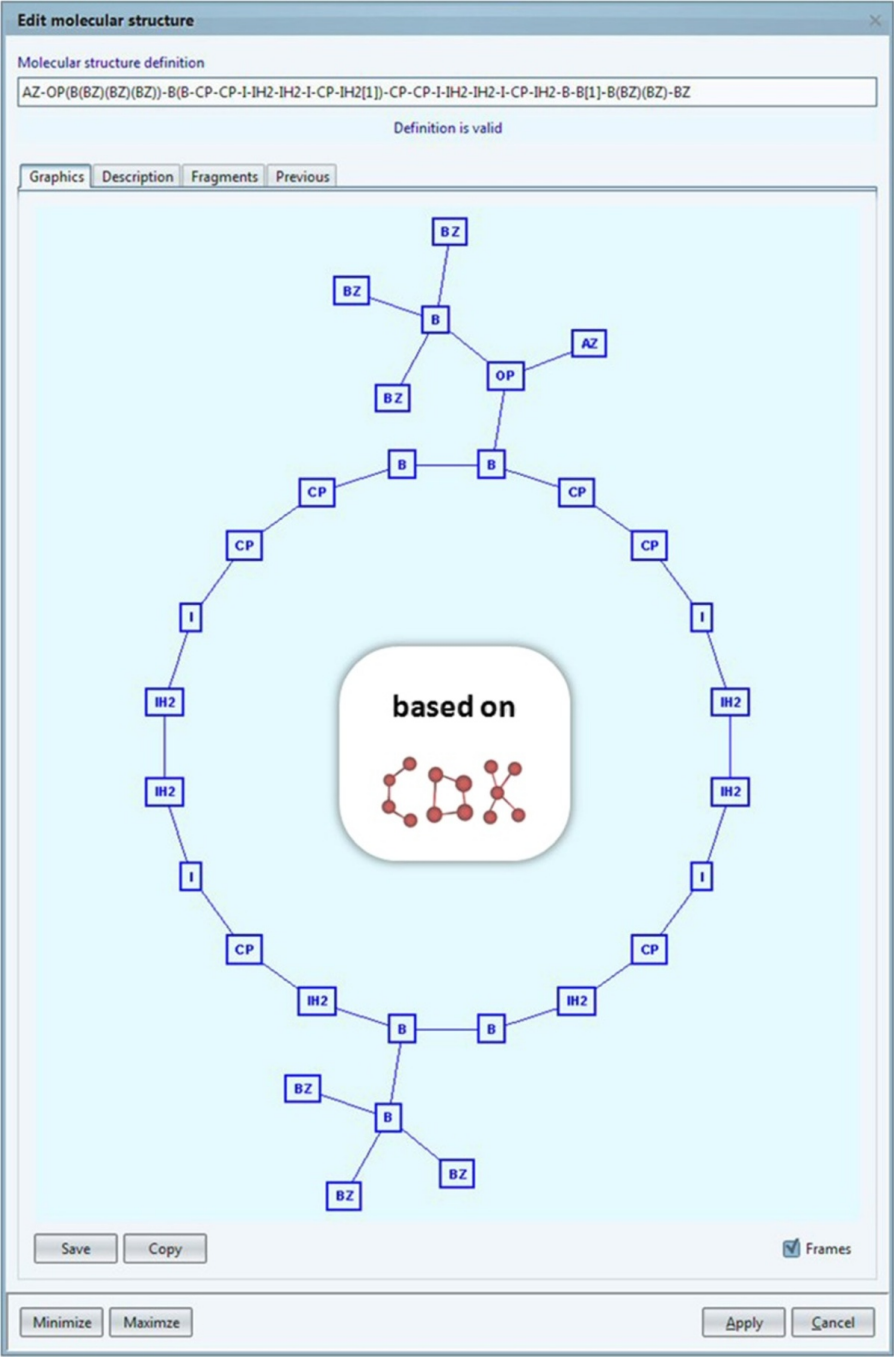
**Fragment diagram layout.** *f*SMILES visualization of a GDNT lipid with a fragment-structure-diagram layout based on the CDK.

There are two different basic types of editors for molecular fragment structures: Textual editors that allow direct *f*SMILES input and graphical editors for drawing of fragment structures, i.e. fragments and their mutual connections. A textual editor can be built with standard text box widgets, a graphical fragment editor could exploit and customize available open-source editors like JChemPaint [27] since a molecular fragment structure consists only of a subset of features of an atom-based molecular structure. A graphical visualization of *f*SMILES could again be achieved by adequate exploitation of open-source projects like the Chemistry Development Kit (CDK) [28],[29]. The structure-diagram layout of the CDK could be customized to display *f*SMILES fragment structures instead of atom-based connection topologies, see Figure 4 for a customized implementation with Java/Swing: The *f*SMILES string is defined in a text box widget, then checked with the *f*SMILES library (see building block 2 below) and finally parsed by CDK-based methods to generate an image with the fragment structure shown in a panel container widget.

#### Fragment structure converters for peptides and proteins

Peptides and proteins consist of distinct sequences of amino acids which fold into secondary and tertiary 3D structures. With an adequate fragment representation for each amino acid and all possible dipeptides, the automated conversion of peptides and proteins into *f*SMILES becomes available.

**Figure 5:**
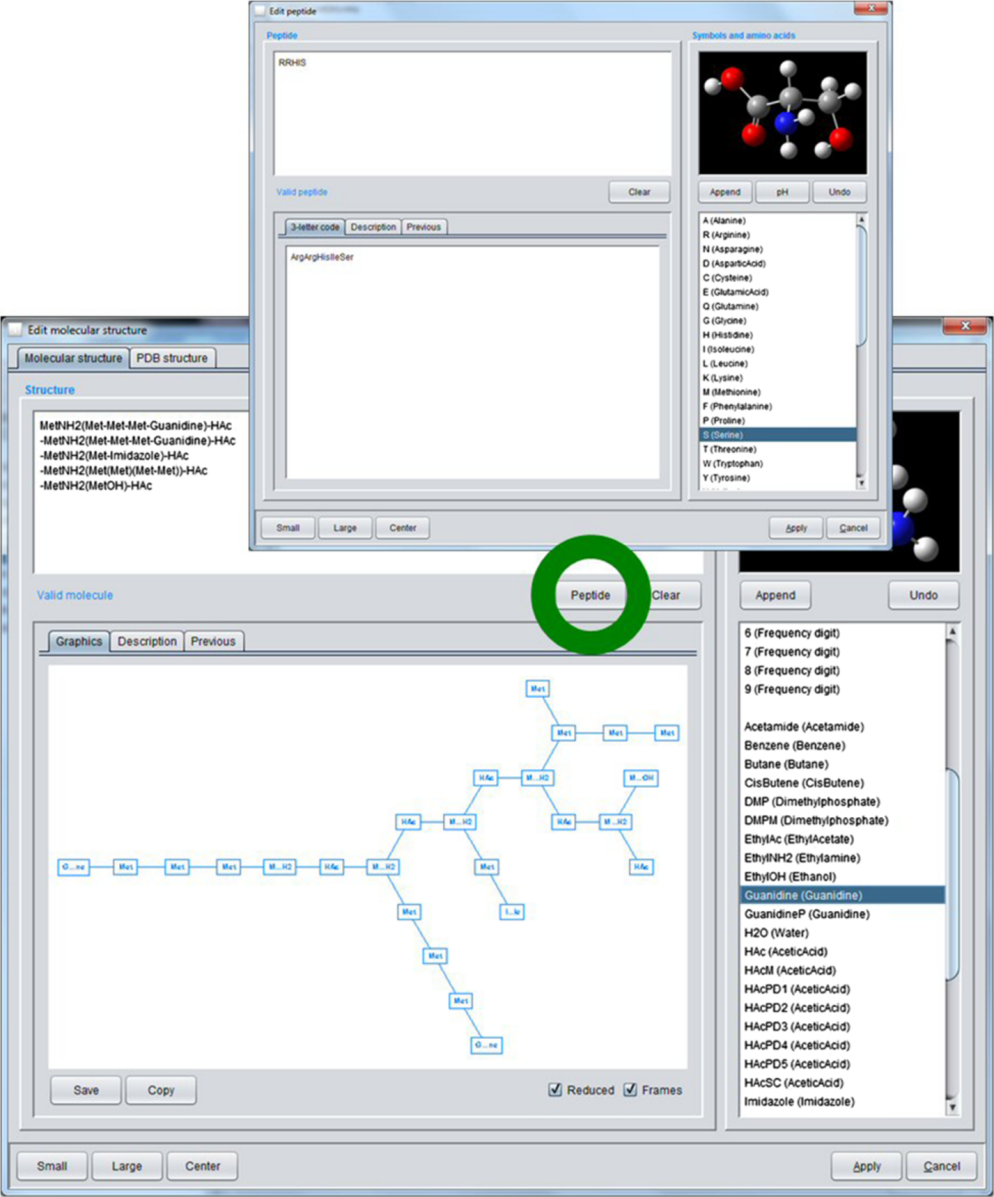
**Peptide editor.** Peptide editor for conversion of a pentapeptide (with one-letter amino acid sequence RRHIS, top small window) into a *f*SMILES representation (bottom large window) and CDK-based structure-diagram layout.





**Figure 6:**
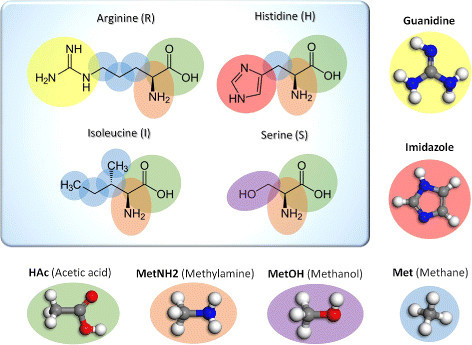
**Amino acid fragmentation.** Fragmentation scheme of selected amino acids.

Figure 5 shows a conversion example of the pentapeptide Arginine-Arginine-Histidine-Isoleucine-Serine (RRHIS) into the corresponding *f*SMILES:

The fragments and the fragmentation scheme for the used amino acids are depicted in Figure 6. Note that the sketched fragmentation scheme does not account for the chiral structure of the proteinogenic amino acids since the methylamine fragment does not contain an asymmetric carbon atom (but an alternative fragmentation scheme may comprise a representative fragment which is a distinct stereoisomer). For an adequate description of the pH-dependent charge state of side-chain and terminal amino acid functional groups, the one-letter code may be extended: For example, at a pH value of 7.4 the pentapeptide RRHIS contains a positively charged amino group at the terminal arginine, two positively charged arginine side chains and a negatively charged carboxylic group at the terminal serine. This could be denoted with additional tags like R{N+S+}R{S+}HIS{C-} where tag {N+} indicates a positively charged terminal amino group of the preceding arginine (R), {S+} indicates a positively charged side chain of the preceding arginine (R) and {C-} the negative charged carboxylic group of the preceding terminal serine (S) respectively.

**Figure 7:**
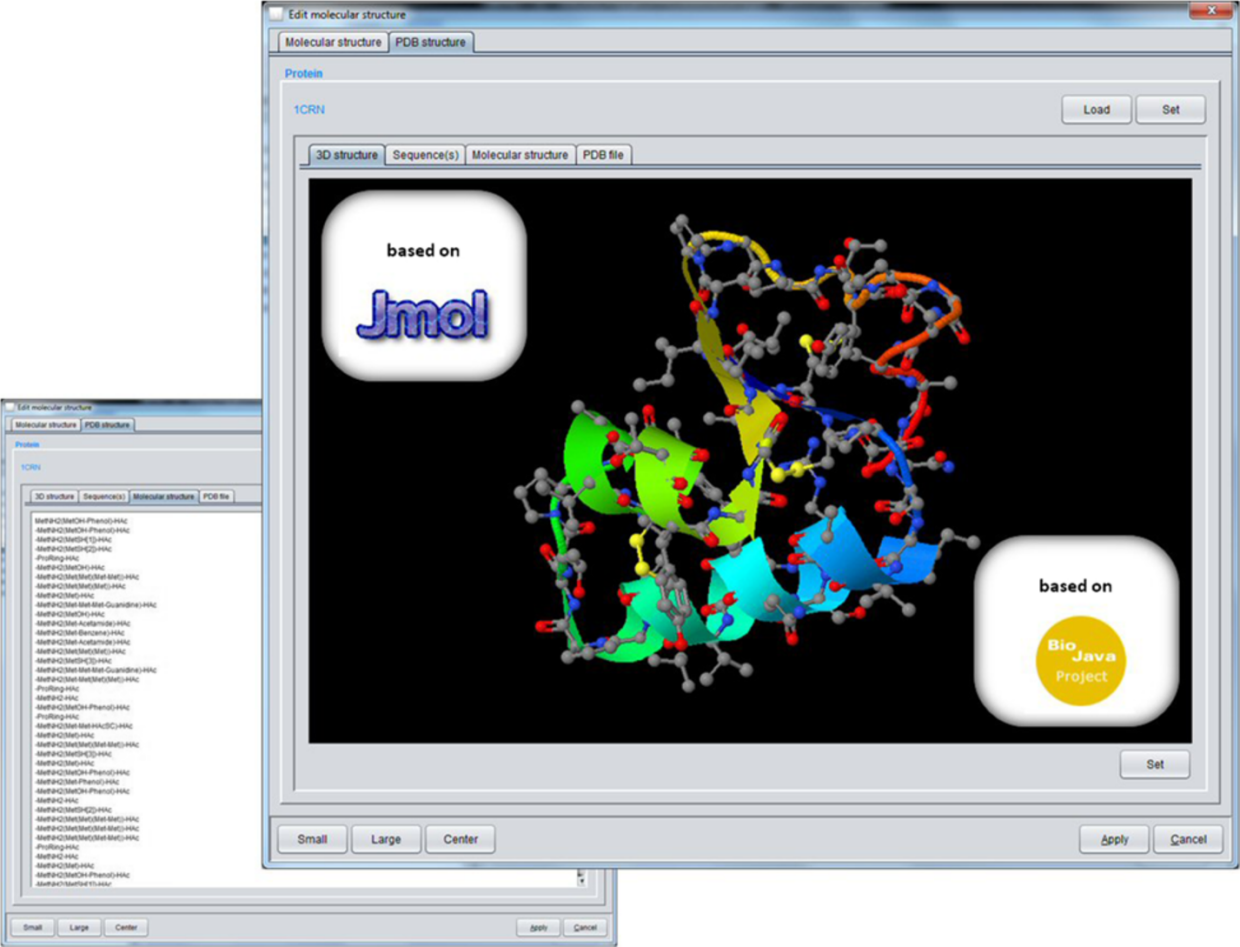
**PDB structure converter.** PDB converter that reads PDB files and converts an amino acid sequence (large window above) into a *f*SMILES representation (small window left).

**Figure 8:**
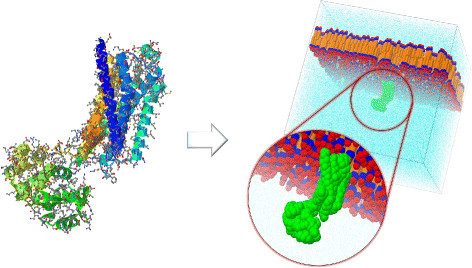
**PDB to MFD: Start geometry of a simulation box (on the right) with a magnified G-protein (cartoon on the left, coloured green on the right) below a phosphatidylethanolamine membrane layer compartment with double-layer orientation (right side).**

For proteins the amino acid sequences can be obtained from Protein Data Bank (PDB) files [30]. These files may be comfortably evaluated and analysed with the open-source library BioJava [31] and graphically displayed with the chemical open-source visualizer Jmol [32] (see Figure 7). Since a PDB file also contains atomic 3D structure coordinates, this spatial information may be used for a corresponding spatial arrangement of the protein backbone fragments inside a sphere compartment (see below and Figure 8).

#### Polymer builder

The simulation of polymers and polymer mixtures is a common application of mesoscopic simulation in general and of MFD in particular. Monomer definition features are already included into the *f*SMILES definition (see above and Appendix A). An additional polymer builder tool could realize tasks like the definition of different statistical monomer distributions within a polymer chain or the creation of specific (alternating, periodic, statistical/random, linear/branched, gradient, block) copolymers (including star/brush/comb structures) by adequate textual construction and manipulation of *f*SMILES strings.

### Building block 2: Operations on fragment structures

In order to make the sketched *f*SMILES fragment structure representation productive, a basic function library is necessary that provides useful operations on these structures.

At first, syntax parser functions are mandatory which allow a detailed syntax check of a provisional *f*SMILES string to ensure its formal correctness. This comprises a left-to-right *f*SMILES string evaluation with separation and translation of every semantic unit (frequencies, fragments, bonds, brackets etc.) into an array of corresponding tokens (where possible forbidden characters or substrings are detected). Then, general checks (like the match of numbers of opening and closing brackets or the pairwise occurrence of ring closures) are performed and finally the token sequence is analysed in a consecutive manner for its syntactic validity.

**Figure 9:**
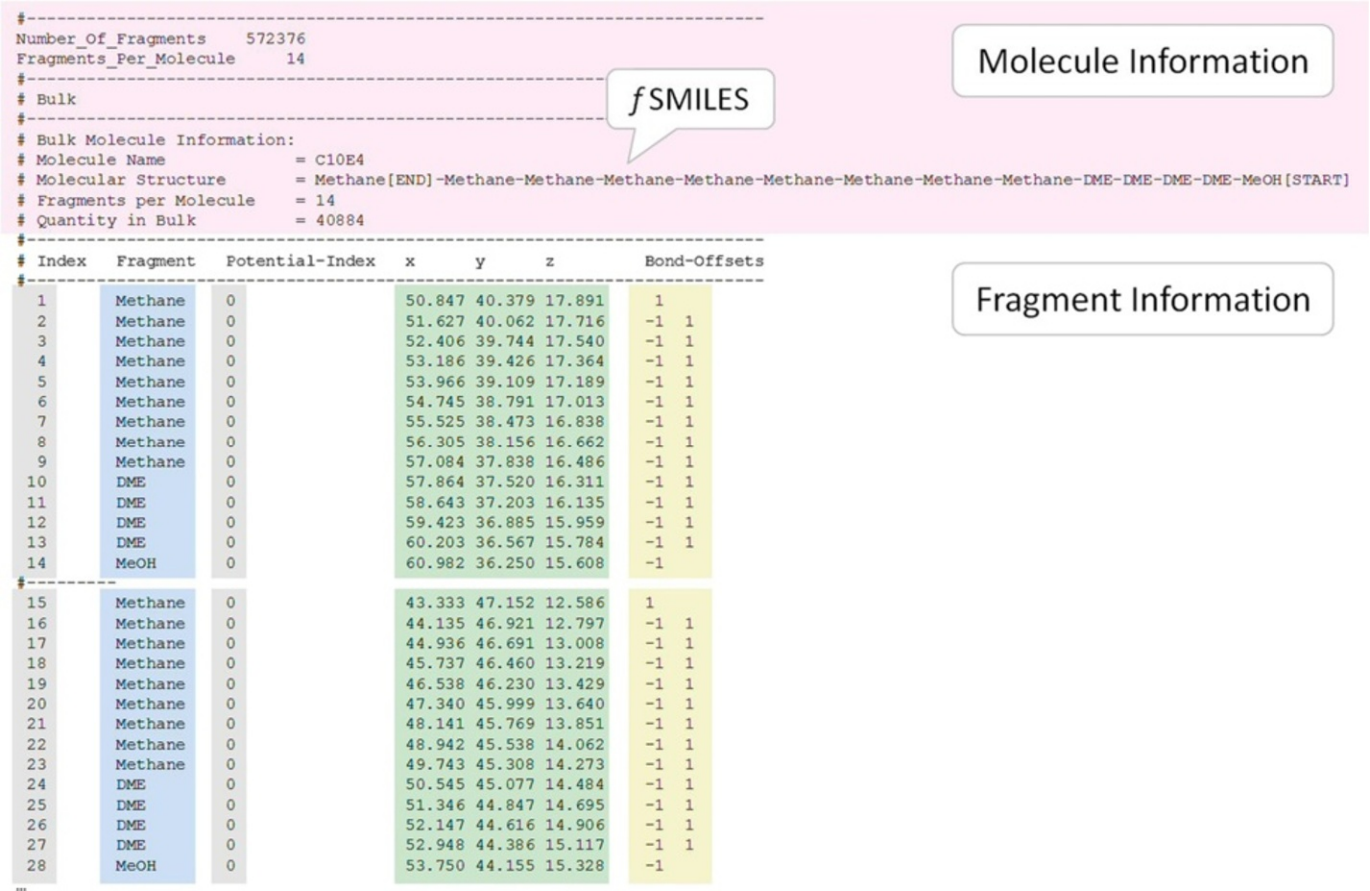
**Molecular start geometry.** Joined result of the *f*SMILES converter and the spatial fragment mapping functions for C _10_*E*_4_ surfactant molecules with *f*SMILES 9Methane-4DME-MeOH9Methane-4DME-MeOH (comment lines start with a “#”).

For communication with the MFD simulation kernel, a converter function is necessary that transforms a valid *f*SMILES into a distinct fragment-connection table/matrix: This function performs the primary man-machine interaction in MFD where the human-comprehensible *f*SMILES string is converted to the machine-readable connection matrix. Since a mesoscopic MFD simulation may comprise thousands to millions of molecules, distinct functions for the initial mapping of their fragments to their spatial box coordinates are necessary to obtain the start configuration of the simulation. Figure 9 illustrates the joined result of the *f*SMILES converter and the spatial fragment mapping functions for C _10_*E*_4_ surfactant molecules with *f*SMILES string 9Methane-4DME-MeOH9Methane-4DME-MeOH in form of an ASCII input file for the MFD kernel that defines the start geometry and connectivity of the first two of 40884 C _10_*E*_4_ molecules in the simulation box. Each C _10_*E*_4_ molecule contains 14 fragments where the position and connectivity of each fragment is described in a single line: The “Index” column attributes an array position to each fragment, followed by the “Fragment” column defining the fragment, a “Potential-Index” column for additional fragment-fragment potentials (e.g. necessary for defining the stiffness of peptide and protein backbones), the “x y z” simulation box coordinates columns and finally relative “Bond-Offsets” for fragment-fragment connections by harmonic springs (where “-1” indicates a connection to the fragment in the previous line and “1” a connection to the fragment in the next line).

The fragment spatial coordinates mapping requires another set of functions that allow an adequate molecule configuration as well as orientation in the simulation box. Since MFD is driven by soft fragment potentials (in contrast to atomic hard core repulsion), different fragments may overlap and even penetrate each other. As a consequence, the problem of possible atomic entanglements or caging effects due to inadequate molecular start geometries is negligible [33]. Nonetheless, a favourable molecular start configuration and orientation may considerably reduce the necessary simulation period. A straightforward approach is a spatial tube representation of the molecular fragment configuration: The longest linear fragment chain in the molecule is determined and its fragments are consecutively lined up along a straight line according to the specified MFD bond length. Then all branched side fragments are collapsed onto their nearest neighbour on this line. For a fast determination of a sufficiently long linear fragment chain, the Depth-First Search (DFS) algorithm may be used [34]: Starting from the first fragment of the *f*SMILES string, the maximum-distant fragment (denoted A) is evaluated by a first DFS run. In a second step, another DFS run is performed to find the maximum-distant fragment from fragment A (denoted B). Finally the fragment chain between fragments A and B is chosen for the spatial tube representation. The sketched method leads to true longest chains for acyclic *f*SMILES but not necessarily for cyclic fragment structures. In the latter case, the determined linear fragment chain is a heuristic result only but still sufficient for all practical purposes. If a “[START]” and an “[END]” fragment are defined (see Appendix A), then the longest linear chain between these tagged fragments is used. If specific additional intramolecular potentials between fragments are defined in order to influence the backbone stiffness of e.g. polymers, peptides or proteins the longest linear chain tube representation may also be regarded as an adequate start configuration for their structural unfolding. For specific large macromolecules like proteins, a known spatial fragment backbone may of course be directly mapped onto the corresponding box coordinates to speed up the simulation (see below and Figure 8).

Last but not least, the function library ought to contain support methods for *f*SMILES-related property calculations like fragment frequencies, monomer-fragment expansion or fragment expansion from frequency counts (as well as their corresponding collapsing methods) and stoichiometric or concentration calculation methods.

### Building block 3: Compartments

**Figure 10:**
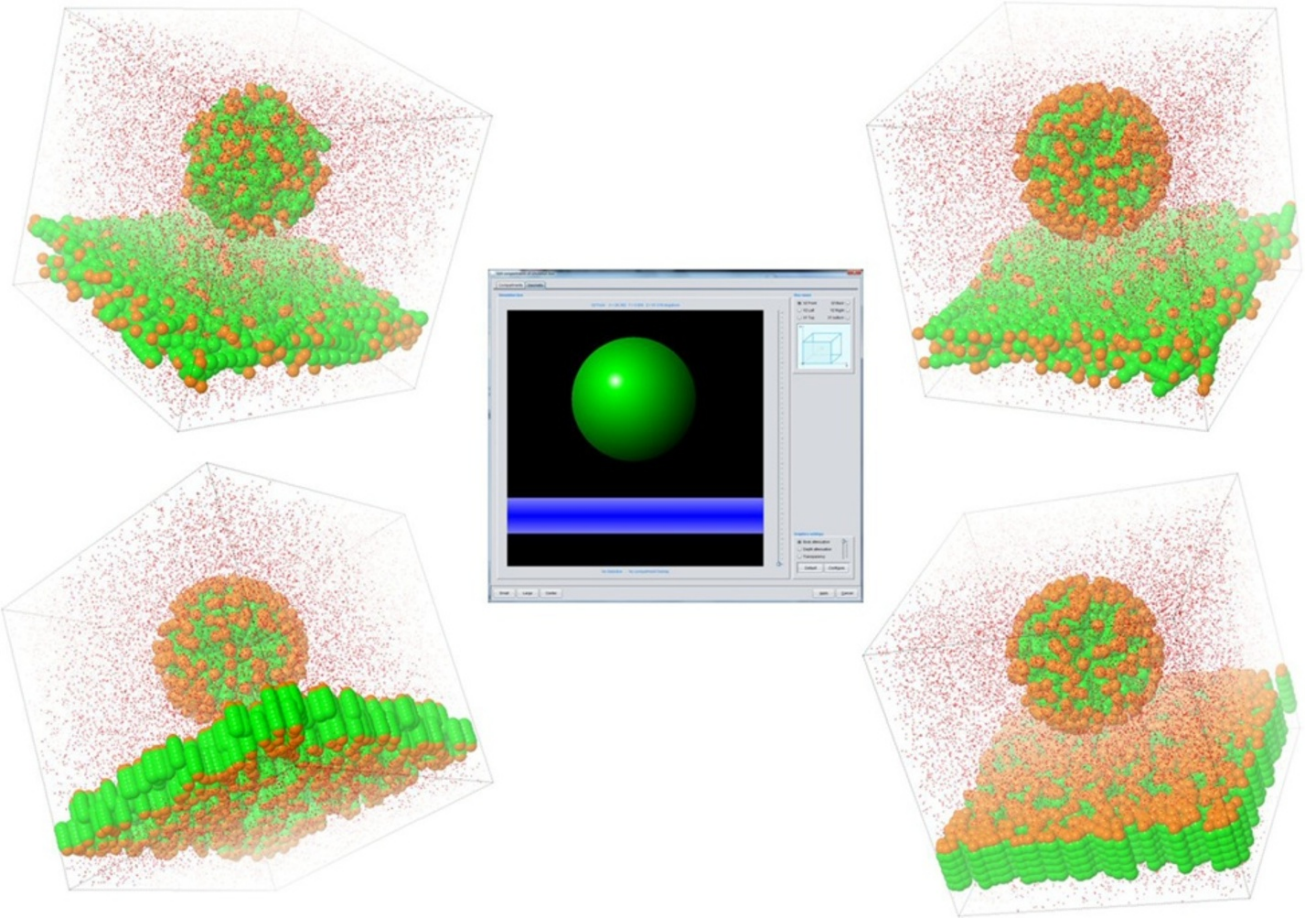
**Compartment definitions in the simulation box with** **B[START]-4A-A[END]** B[START]-4A-A[END] **molecules in spatial tube representation (fragments A in green, fragments B in orange, bulk fragments are scaled down in red.** Center window: Compartment editor; upper left: Layer and sphere compartment with random molecule positions, upper right: Sphere compartment with radial molecule orientation and random layer compartment, lower left: Sphere compartment with radial molecule orientation and layer compartment with double layer orientation, lower right: Sphere compartment with radial molecule orientation and layer compartment with single layer orientation.

Mesoscopic simulation targets large molecular ensembles which represent up to millions of molecules. The start geometry of the simulation box may be a pure random bulk mixture with molecules represented by spatial tubes (see above). But in many cases, this global “random soup” is unfavourable for a specific simulation. To allow for a more detailed spatial setting of the start configuration, the definition of simulation box compartments is mandatory. Common compartment types are spheres, layers or bricks which may be freely positioned in the simulation box with or without overlap. The size of the compartments determines the number of fragments it may contain. Each compartment may possess a specific molecular composition as well as specific orientation and alignment of its molecules/fragments. Figure 10 shows a simulation box compartment editor and corresponding boxes with different molecular orientation and alignment where the compartments are filled with B[START]-4A- A[END] molecules (fragments A in green, fragments B in orange). The molecular spatial tubes may be randomly distributed in a compartment (Figure 10, upper left) or aligned in a specific manner, e.g. with a radial orientation in a sphere compartment with the orange “[START]” fragment at the surface (Figure 10, upper right). In layer departments, the molecular spatial tubes may be aligned in single or double layers (Figure 10, lower left/right). To model solid surfaces, the fragments of a layer compartment may be positioned according to crystal structures like a simple cubic lattice. The remaining simulation box bulk volume is usually filled up in a random manner as a satisfactory default. Macromolecules like proteins with distinct 3D backbone geometries can be scaled within a sphere compartment: Whereas the protein backbone fragments are still in proper spatial relations to each other derived from a corresponding PDB file (but usually “a bit squeezed” into the sphere), the amino acid side chain fragments are collapsed onto their neighbour fragments. As an example, the start geometry of a simulation box with a “squeezed” G-protein sphere compartment (PDB ID: 2RH1 [35], colored green) in a sphere compartment below a phosphatidylethanolamine membrane compartment (right side) is shown in Figure 8. The spatial configuration of the protein backbone fragments is derived from the crystal structure of the PDB structure on the left (both rendered with Jmol, see below). With the sketched compartment approach, a wide range of molecular start geometries may be constructed with acceptable effort.

### Building block 4: Simulation box

The graphical visualization of the simulation box, i.e. the fragments at their box positions, is a central feature of every molecular simulation method. Mesoscopic MFD simulation box visualization in particular is challenged by its large size with up to millions of fragments. On the other hand the simulation box visualization is confined to adequate 3D spherical fragment rendering only since fragment connections are usually omitted without any loss of display quality. A simulation box visualization should allow arbitrary box rotations, specific fragment/molecule size scaling up to their exclusions, arbitrary re-colouring of fragments, zoom in/out functions as well as length measurement options. Last but not least the creation of simulation box movies that comprise reasonable simulation steps should be supported.

**Figure 11:**
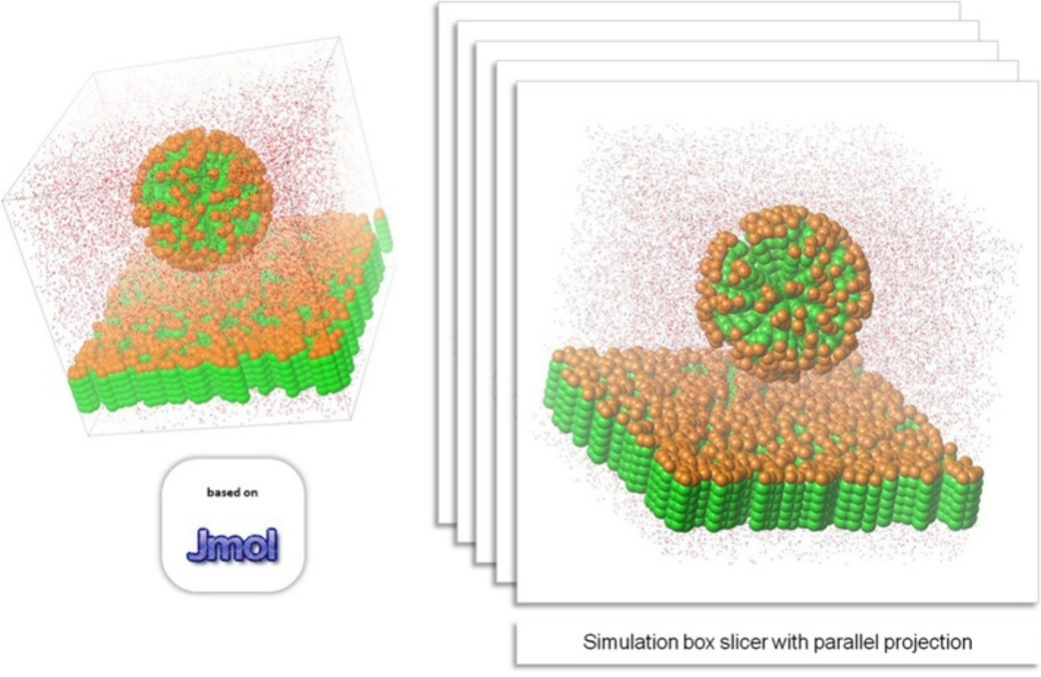
**Simulation box visualization.** Simulation box display and manipulation, left: Jmol based box, right: Corresponding simulation box slicer with parallel projection.

One approach is the use of established open-source atom-based molecular visualization tools like Jmol [32] which can be customized in a programmatic manner by adequate scripts: If the MFD fragments are mapped onto Jmol atom types, all visualization functions of Jmol are available, e.g. the box displays of Figures 8, 10 and 11 (on the left) are generated with a customized Jmol implementation.

For minimum memory consumption and very fast rendering, a simulation box slicer approach may be followed alternatively: The simulation box is cut into slices along an axis of interest and the slice graphics are rendered one by one which leads to a graphical illustration with parallel projection that alleviates through-space length comparisons and measurements. For fragment rendering a fast 2D radial-gradient paint may be used to create a 3D sphere illusion. The depth impression of the simulation box may be adjusted by a fog option. Figure 11 (on the right) demonstrates a result of this approach. From corresponding box slice views of different simulation steps a simulation movie can be created with little effort.

## Conclusions

A consequent MFC roadmap with an adequate fragment structure representation (*f*SMILES), defined operations on these fragment structures, the description of compartments with defined compositions and structural alignments and the setup and analysis of a whole simulation box may considerably alleviate and broaden the use of mesoscopic simulation techniques like MFD. In addition the MFC roadmap realization can draw an already existing Cheminformatics open-source solutions like CDK, BioJava or Jmol. Thus, MFC may be regarded as a crucial accelerator to propagate MFD and similar mesoscopic simulation techniques in the molecular sciences. The MFC layer itself may then be utilized by a graphical user interface (GUI) approach to finally build a rich simulation client for convenient practical application even in industrial environments. Last but not least, MFC creates new cheminformatics challenges like a computer-assisted/automated fragmentation support to split a chemical compound of interest into adequate molecular fragments.

## Appendix A - Molecular fragment structure definition

The following rules allow the definition of a monomer or molecular fragment structure line notation (*f*SMILES):

1. Fragment names with a maximum of 10 characters (a-z, A-Z, 0-9, first non-digit) and an optionally prefixed frequency number.
2. Connection character ‘-’ for bonding of the main chain.
3. Round brackets ’(’ and ’)’ indicating branches. They may be nested for arbitrary levels of branches.
4. Square brackets ’[’ and ’]’ with an enclosed number which follow a fragment indicating a ring closure.
5. Curly brackets ’{’ and ’}’ with an enclosed monomer definition. Monomers are defined as molecular structures but must contain at least 1 fragment with a [HEAD] and [TAIL] attribute: Structure elements that precede the monomer connect to the HEAD fragment, structure elements that follow the monomer connect to the TAIL fragment. Monomers are not allowed to be nested.
6. Monomer labels that start with a ‘#’ character followed by a sequence of characters (first non-digit).
7. Monomer labels may be preceded by a frequency number.
8. A fragment of a molecular structure (but not of a monomer) may optionally contain a [START] or an [END] tag which may be used for orientation purposes. There is only one [START]/[END] pair allowed per structure.
9. A molecule may consist of multiple independent parts (i.e. parts are not allowed to be connected in any way). Each part must be framed by angle brackets ‘<’ and ‘>’. Parts are not allowed to be nested.

## Appendix B - Molecular fragment structure examples

- The following examples demonstrate the usage of the *f*SMILES definitions sketched in Appendix A:
- A-B-CA-B-C defines a connection of fragment A with fragment B and fragment B with fragment C.
- A-2B(E-F)-DA-2B(E-F)-D is identical to A-B-B(E-F)-DA-B-B(E-F)-D.
- 3A-B3A-B is a shortcut notation for A-A-A-BA-A-A-B.
- A-B(D-E)-FA-B(D-E)-F defines a main chain A-B-FA-B-F with a side chain D-ED-E where fragment D is connected to fragment B.
- A-B[1]-C-C-C-D-E[1]A-B[1]-C-C-C-D-E[1] defines a ring closure between fragments B and E.
- A-B(D-E(G-H[1])-F)-I-A-K[1]-BA-B(D-E(G-H[1])-F)-I-A-K[1]-B defines a main chain A-B-I-A-K-BA-B-I-A-K-B with a side chain D-E-FD-E-F (connected to fragment B of the main chain) and another side chain G-HG-H (connected to fragment E of the first side chain). In addition there is a ring closure between fragment H of the second side chain and fragment K of the main chain.
- 3A[1]-B-B-C[1]3A[1]-B-B-C[1] is a shortcut for A-A-A[1]-B-B-C[1]A-A-A[1]-B-B-C[1].
- 3A(B)-D3A(B)-D is a shortcut for A-A-A(B)-DA-A-A(B)-D.
- Multiple ring closures at one fragment are marked by successive use of ring-closure brackets, e.g. fragment B in A-B[1][2]-4C-D[1]-4C-E[2]A-B[1][2]-4C-D[1]-4C-E[2] is connected to fragments D and E.
- The simplest structure of a monomer consists of a single fragment A with attributes [HEAD] and [TAIL], i.e. {A[HEAD][TAIL]}{A[HEAD][TAIL]}.
- D-#MyMonomer-FD-#MyMonomer-F with #MyMonomer equal to {A[HEAD]-B-B[TAIL]-C}{A[HEAD]-B-B[TAIL]-C} defines a structure where fragment D is connected to fragment A (the head) of the monomer and Fragment B (the tail) of the monomer is connected to fragment F. This definition is equivalent to D-A-B-B(C)-FD-A-B-B(C)-F.
- 2{A[HEAD]-B-B[TAIL]-C}2{A[HEAD]-B-B[TAIL]-C} defines a structure of 2 monomers where fragment A (the head) of the second monomer is connected to Fragment B (the tail) of the preceding first monomer. This definition is equivalent to A-B-B(C)-A-B-B-CA-B-B(C)-A-B-B-C.
- Definitions like {A[HEAD]-{A[HEAD]-B-B[TAIL]-C}-B[TAIL]-C}{A[HEAD]-{A[HEAD]-B-B[TAIL]-C}-B[TAIL]-C} with nested monomers are forbidden.
- A[START]-B-C[END]A[START]-B-C[END] defines orientation information.
- A[START][END]-B-CA[START][END]-B-C is syntactically correct but makes no sense.
- A[START]-B[START]-C[END]A[START]-B[START]-C[END] is forbidden: There is only one [START]/[END] pair allowed per structure.
- 3A[START]-B-C[END]3A[START]-B-C[END] is identical to A-A-A[START]-B-C[END]A-A-A[START]-B-C[END].
- <A-B-C><A-D><A-B-C><A-D> defines a molecule which consists of two independent parts A-B-CA-B-C and A-DA-D.
- <A-B[1]-C><A-D[1]><A-B[1]-C><A-D[1]> is forbidden since parts are not allowed to be connected in any way. The correct definition in this case would be (A-B[1]-C)(A-D[1])(A-B[1]-C)(A-D[1]) or A-B(C)-D-AA-B(C)-D-A.

## Authors’ original submitted files for images

Below are the links to the authors’ original submitted files for images. Authors’ original file for figure 1 Authors’ original file for figure 2 Authors’ original file for figure 3 Authors’ original file for figure 4 Authors’ original file for figure 5 Authors’ original file for figure 6 Authors’ original file for figure 7 Authors’ original file for figure 8 Authors’ original file for figure 9 Authors’ original file for figure 10 Authors’ original file for figure 11 Authors’ original file for figure 12 Authors’ original file for figure 13
